# Endothelial Cell-Derived Soluble CD200 Determines the Ability of Immune Cells to Cross the Blood–Brain Barrier

**DOI:** 10.3390/ijms25179262

**Published:** 2024-08-27

**Authors:** Myriam Pujol, Tautvydas Paskevicius, Alison Robinson, Simran Dhillon, Paul Eggleton, Alex S. Ferecskó, Nick Gutowski, Janet Holley, Miranda Smallwood, Jia Newcombe, Luis B. Agellon, Marek Michalak

**Affiliations:** 1 Department of Biochemistry, University of Alberta, Edmonton, AB T6G 2H7, Canada; pujolmor@ualberta.ca (M.P.); tautvyda@ualberta.ca (T.P.); at24@ualberta.ca (A.R.); sk2@ualberta.ca (S.D.); 2 Revolo Biotherapeutics, Gaithersburg, MD 20878, USA; p.eggleton@exeter.ac.uk; 3 University of Exeter Medical School, University of Exeter, Exeter EX1 2HZ, UK; alex.ferecsko@ucb.com (A.S.F.); n.j.gutowski@exeter.ac.uk (N.G.); j.e.holley@exeter.ac.uk (J.H.); m.j.smallwood@exeter.ac.uk (M.S.); 4 NeuroResource, Department of Neuroinflammation, UCL Queen Square Institute of Neurology, University College London, London WC1E 6BT, UK; j.newcombe@ucl.ac.uk; 5 School of Human Nutrition, McGill University, Sainte Anne de Bellevue, QC H9X 3V9, Canada

**Keywords:** brain endothelial cells, calnexin, fatty acid binding protein 5, CD200, sCD200, CD200R1, neurodegenerative diseases

## Abstract

The infiltration of immune cells into the central nervous system mediates the development of autoimmune neuroinflammatory diseases. We previously showed that the loss of either Fabp5 or calnexin causes resistance to the induction of experimental autoimmune encephalomyelitis (EAE) in mice, an animal model of multiple sclerosis (MS). Here we show that brain endothelial cells lacking either Fabp5 or calnexin have an increased abundance of cell surface CD200 and soluble CD200 (sCD200) as well as decreased T-cell adhesion. In a tissue culture model of the blood–brain barrier, antagonizing the interaction of CD200 and sCD200 with T-cell CD200 receptor (CD200R1) via anti-CD200 blocking antibodies or the RNAi-mediated inhibition of CD200 production by endothelial cells increased T-cell adhesion and transmigration across monolayers of endothelial cells. Our findings demonstrate that sCD200 produced by brain endothelial cells regulates immune cell trafficking through the blood–brain barrier and is primarily responsible for preventing activated T-cells from entering the brain.

## 1. Introduction

Neuroinflammation is mediated by infiltration of the brain by immune cells that cross the blood–brain barrier [1,2]. Immune cell infiltration of the central nervous system (CNS) results in the induction of inflammatory mediators that are associated with autoimmune inflammatory diseases manifested by the progressive demyelination of axons in the CNS [3,4,5]. CD200, an immune checkpoint molecule, is a transmembrane glycoprotein and a member of the immunoglobulin protein superfamily that is broadly expressed on cells of the CNS, including neurons, astrocytes, oligodendrocytes, and endothelial cells [6,7]. The interaction of CD200 with the CD200 receptor 1 (CD200R1) expressed on leukocytes and microglia triggers a regulatory signaling cascade that suppresses local inflammation and promotes a resting state in immune cells [8,9,10]. Brain infiltration by myeloid and lymphoid cells is highly increased in CD200-deficient mice [11], which indicates the importance of this protein in maintaining blood–brain barrier permeability for leukocytes [2,3,4,12].

We previously reported that the loss of calnexin leads to peripheral axon demyelination [13]. Calnexin is an endoplasmic reticulum (ER)-resident protein that is commonly referred to as a molecular chaperone [14]. In search of calnexin interaction partners, we discovered that the cytosolic C-tail domain of calnexin makes stable protein–protein interaction with a cytosol-resident protein known as fatty acid binding protein 5 (Fabp5) [15] to form a complex at the cytosol–ER interface [16,17]. Remarkably, both calnexin-deficient and Fabp5-deficient mice are resistant to the induction of experimental autoimmune encephalomyelitis (EAE) [17,18,19,20], an animal model of CNS inflammation including multiple sclerosis (MS) [21,22]. EAE pathogenesis exhibits many of the hallmarks of neuroinflammatory autoimmune disorder, including the ability of autoreactive T-cells to traverse the blood–brain barrier with consequent CNS cell infiltration [21,22], and our studies indicate that the resistance of *Canx*^−/−^ mice to EAE induction is attributable to the limited ability of T-cells to cross brain endothelial cells [17,20]. Furthermore, we found that the formation of the Fabp5/calnexin complex [16] is a prerequisite for the sensitization of mice to EAE induction [20].

The naturally occurring mouse mutant known as the Wallerian degeneration (*Wld^S^*) mouse has been reported to display resistance to EAE [23], similar to *Canx*^−/−^ and *Fabp5*^−/−^ mice [17,20]. Interestingly, *Wld^S^* mice were shown to overproduce the neuroprotective protein CD200 [23]. Hence, here we tested the hypothesis that CD200 is involved in regulating the transmigration of activated T-cells through the blood–brain barrier.

## 2. Results

**Figure 1:**
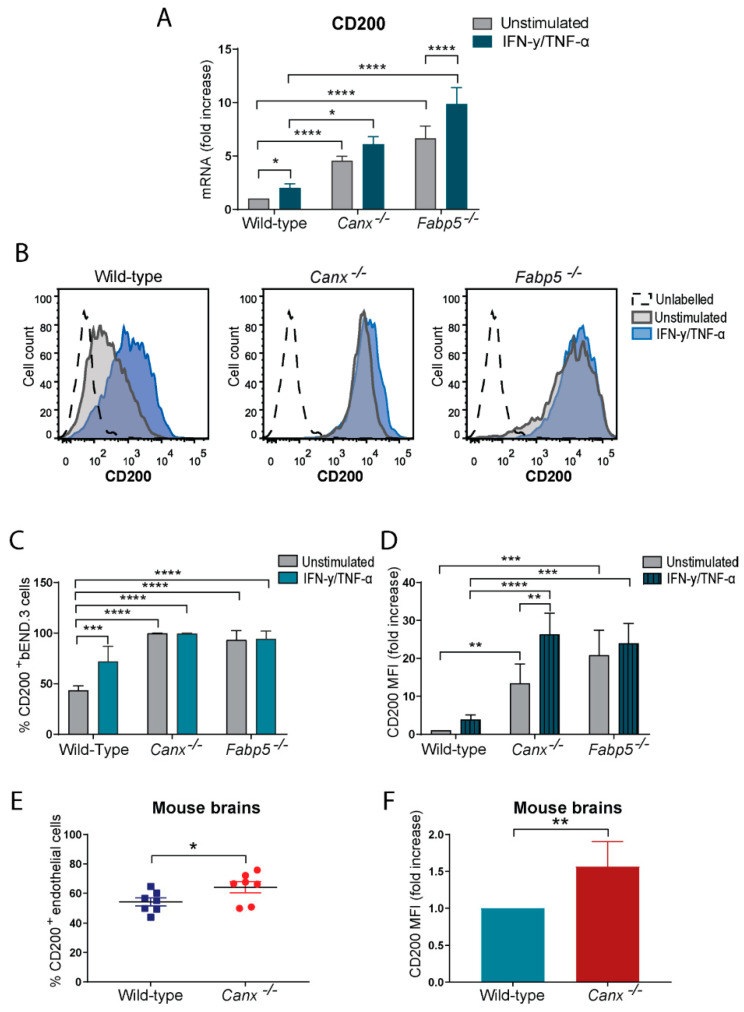
CD200 on brain endothelial cells. (**A**) Relative quantification shows higher CD200 mRNA abundance in *Canx^−/−^* or *Fabp5^−/−^* cells compared to wild-type cells (based on 7 experiments performed in duplicates). (**B**) Representative histograms of negative control (dashed line), unstimulated cells (grey histogram), and cytokine stimulated cells (blue histogram). (**C**) CD200^+^ cells in wild-type, *Canx^−/−^*, and *Fabp5^−/−^* bEND.3 cell populations. (**D**) Cell surface abundance (mean fluorescence intensity, MFI) of CD200 on unstimulated and cytokine-treated wild-type, *Canx^−/−^*, and *Fabp5^−/−^* cells. (**E**) Percentage of CD200 in CD11b^−^PECAM-1^+^ endothelial cells isolated from brains of *Canx^−/−^* and wild-type mice. (**F**) MFI values of gated CD200^+^CD11b^−^PECAM-1^+^ brain endothelial cells. All data shown are from 7 independent experiments. * *p* < 0.05, ** *p* < 0.01, *** *p* < 0.001, **** *p* < 0.0001.

CD200 abundance is increased in *Canx^−/−^* or *Fabp5^−/−^* bEND.3 cells and *Canx^−/−^* mouse brain endothelial cells. *Canx^−/−^* or *Fabp5^−/−^* mice are resistant to EAE [17,20]. *Wld^S^* mice also display an attenuated course of EAE development with a delayed onset of the disease accompanied by a large increase in the abundance of CD200 in the CNS [23], whereas the induction of EAE in wild-type mice is accompanied by the decreased abundance of CD200 [24]. We therefore examined whether CD200 is also associated with the EAE resistance phenotype of *Canx^−/−^* and *Fabp5^−/−^* mice [17,20] and, in particular, if cell surface CD200 expression was altered in cytokine-stimulated bEND.3 cells that were deficient in either calnexin or Fabp5. First, Q-PCR analysis revealed an increase in the abundance of CD200 mRNA in *Canx^−/−^* and *Fabp5^−/−^* bEND.3 cells (Figure 1A). Importantly, flow cytometry analysis showed a remarkable increase in the cell surface abundance of CD200 in both *Canx^−/−^* and *Fabp5^−/−^* bEND.3 cells compared to their wild-type counterpart regardless of whether they were stimulated with cytokines or not (Figure 1B–D,). Incredibly, 99.6% of unstimulated *Canx^−/−^* and 93.1% of *Fabp5^−/−^* bEND.3 cells were CD200^+^*,* compared to only 37.8% of wild-type cells (Figure 1B,C). Furthermore, *Canx^−/−^* and *Fabp5^−/−^* bEND.3 cells had 13-fold (*p* = 0.003) and 20-fold (*p* = 0.0002) higher cell surface CD200 MFI over wild-type bEND.3 cells in the absence of cytokine stimulation (Figure 1D). Upon cytokine stimulation, cell surface CD200 MFI increased further, 26-fold (*p* < 0.0001) in *Canx^−/−^* bEND.3 cells and 24-fold (*p* < 0.001) in *Fabp5^−/−^* bEND.3 cells, but it was not significantly increased in stimulated wild-type bEND.3 cells (3.9-fold, *p* = 0.5432) (Figure 1D). Furthermore, the flow cytometry analysis of *Canx^−/−^* mouse brain tissue revealed a significant increase in CD200^+^ brain endothelial cells (CD11b^−^PECAM-1^+^) (Figure 1E).

### 2.1. CD200 Regulates T-Cell Transmigration across Canx^−/−^ or Fabp5^−/−^ bEND.3 Cell Monolayers

**Figure 2:**
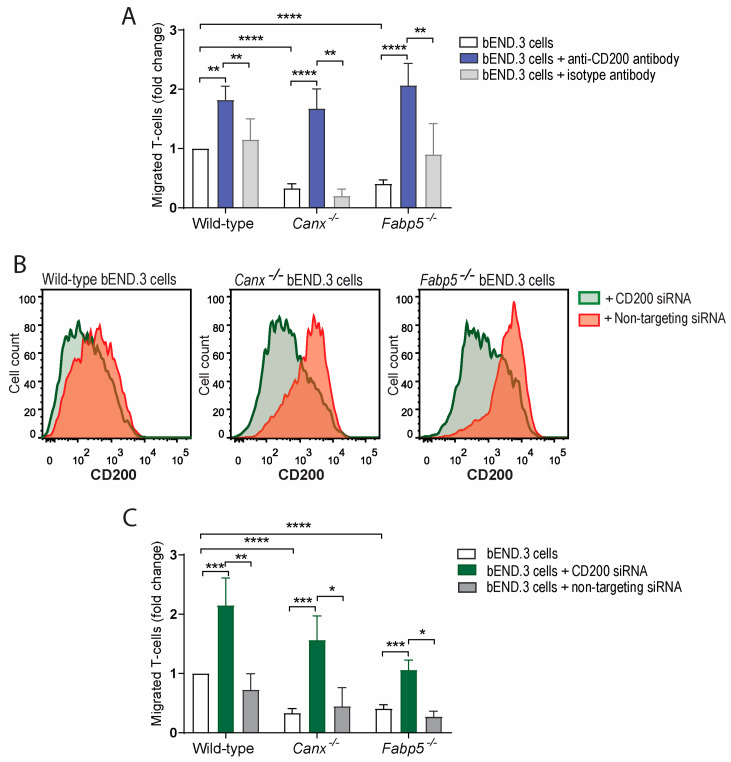
Transmigration of activated T-cells across wild-type, *Canx^−/−^*, or *Fabp5^−/−^* bEND.3 cell monolayers. (**A**) Percentage of activated T-cells migrated through *Canx^−/−^* or *Fabp5^−/−^* bEND.3 cell monolayers vs. wild-type bEND.3 cell monolayers (white bars). The use of anti-CD200 blocking antibody increased T-cell transmigration across wild-type, *Canx^−/−^*, or *Fabp5^−/−^* bEND.3 cell monolayers (blue bars), while the use of the control isotype antibody did not (grey bars). (**B**) Representative histograms of wild-type, *Canx^−/−^*, or *Fabp5^−/−^* bEND.3 cells treated with CD200 siRNA and non-targeting siRNA. (**C**) The siRNA-mediated downregulation of CD200 in wild-type, *Canx^−/−^*, or *Fabp5^−/−^* bEND.3 cell monolayers also increased the transmigration of T-cells across wild-type, *Canx^−/−^*, and *Fabp5^−/−^* bEND.3 cell monolayers. Data representative of 8 independent experiments analyzed in triplicates. * *p* < 0.05, ** *p* < 0.01, *** *p* < 0.001, **** *p* < 0.0001.

Since CD200 plays an important role in protecting immunologically privileged tissues from immune cell infiltration [25], we evaluated the role of CD200 in T-cell transmigration across our established tissue culture model of the blood–brain barrier [17,20]. As we have shown previously, the loss of either calnexin or Fabp5 in bEND.3 cells suppressed the transmigration of activated T-cells across the model blood–brain barrier (*p* < 0.0001 compared to wild-type) [17,20]. Remarkably, the treatment of monolayers comprised of either *Canx^−/−^* or *Fabp5^−/−^* bEND.3 cells with anti-CD200 blocking antibody permitted T-cell transmigration across the monolayers (*p* < 0.0001) to the same extent as monolayers of wild-type bEND.3 cells (Figure 2A). To verify that the permissibility of T-cells to traverse monolayers of bEND.3 cells was due to the antagonism of CD200/CD200R interaction, we used a siRNA-mediated approach to decrease CD200 gene expression, and in particular the cell surface abundance of CD200 in bEND.3 cells. Consistently with the outcome of anti-CD200 blocking antibody experiments (Figure 2A), the siRNA-dependent inhibition of CD200 expression resulted in increased T-cell transmigration across *Canx^−/−^* or *Fabp5^−/−^* bEND.3 cells monolayers (*p* = 0.0009 and *p* = 0.002, respectively; Figure 2B,C). These findings demonstrate that CD200 gene expression by endothelial cells modulates T-cell transmigration across the blood–brain barrier.

In CNS inflammatory response, cell adhesion is followed by leukocyte transmigration through the endothelial layer of the blood–brain barrier [12]. Therefore, we examined whether an altered abundance of adhesion molecules and tight junction proteins contributed to the inability of T-cells to bind to brain endothelial cells and mediate leukocyte transmigration. Flow cytometry analysis showed that there was a comparable increase in the abundance of ICAM-1 and VCAM-1 leukocyte adhesion markers as well as of JAM-A tight junction protein in wild-type, *Canx^−/−^,* and *Fabp5^−/−^* bEND.3 cells stimulated with cytokines (TNF-α/IFN-γ) (Supplementary Figure S1), indicating there was no lack of the cell surface presence of these proteins. These findings indicate that the loss of either calnexin or Fabp5 did not significantly influence the abundance of these adhesion markers on bEND.3 endothelial cells, nor their remodeling upon cytokine treatment.

### 2.2. Soluble CD200 (sCD200) Antagonizes T-Cell Interaction with bEND.3 Brain Endothelial Cells

**Figure 3:**
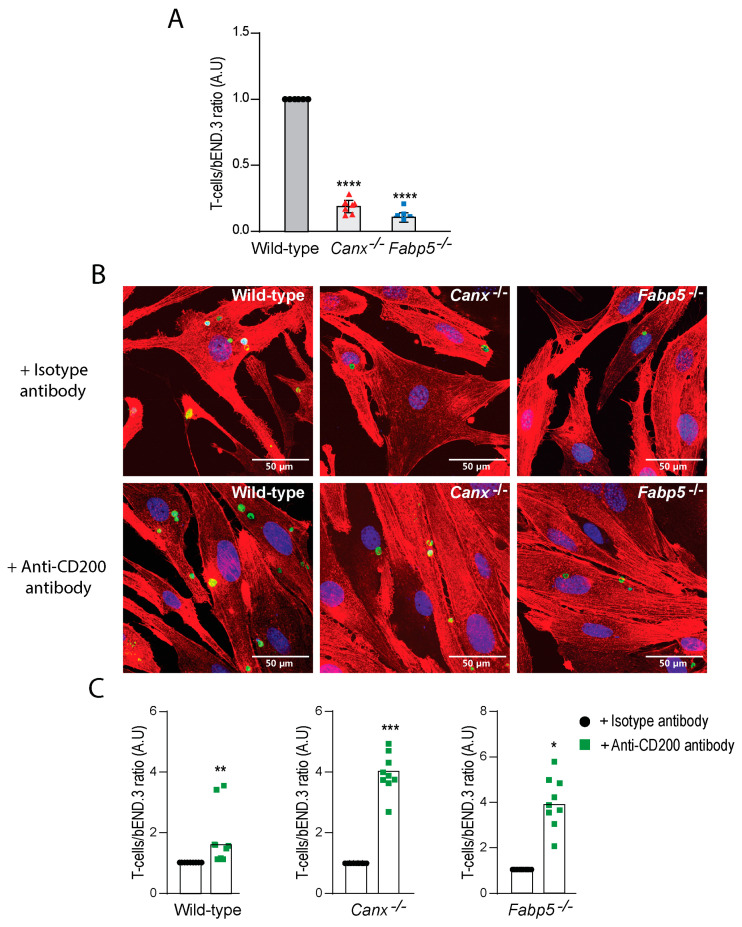
CD200-dependent binding of T-cells to wild-type, *Canx^−/−^*, and *Fabp5^−/−^* bEND.3 cells. (**A**) Relative quantification of T-cells adhered to wild-type, *Canx^−/−^*, and *Fabp5^−/−^* bEND.3 cells. (**B**) Representative images of T-cells (green) adhered to wild-type, *Canx^−/−^*, and *Fabp5^−/−^* bEND.3 cells (red). (**C**) Increased T-cell adherence to wild-type, *Canx^−/−^*, and *Fabp5^−/−^* bEND.3 cells in the presence of anti-CD200 antibodies. * *p* = 0.0325, ** *p* = 0.006, *** *p* = 0.0011, **** *p* < 0.0001. Data shown are the combined results of 3 experiments performed in triplicates. Blue, DAPI staining.

The increased abundance of CD200 on the surface of *Canx*^−/−^ or *Fabp5*^−/−^ bEND.3 cells, as revealed by flow cytometry analysis, suggested that its interaction with CD200R on T-cells may result in their entrapment on endothelial cell surfaces, thus preventing transmigration. To examine this possibility, we analyzed the adhesion of T-cells to bEND.3 cells by confocal microscopy. Unexpectedly, endothelial cells lacking either calnexin or Fabp5 demonstrated substantially less T-cell adhesion (*p* < 0.0001) (Figure 3A), while notably, the addition of anti-CD200 blocking antibody to the culture medium of these cells increased the number of adherent T-cells (wild-type, *p* = 0.0154; *Canx^−/−^, p* = 0.0001; *Fabp5^−/−^, p* = 0.0047) (Figure 3B,C).

**Figure 4:**
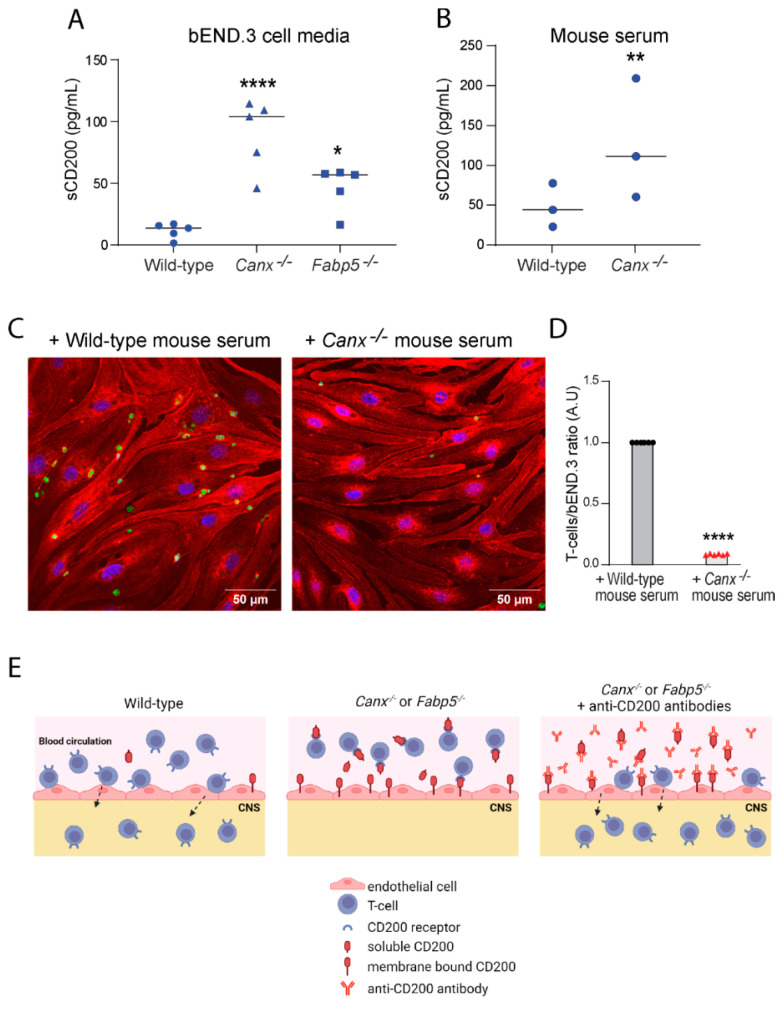
Endothelial cell-derived soluble CD200. (**A**) Soluble CD200 (sCD200) in conditioned media of wild-type, *Canx^−/−^*, or *Fabp5^−/−^* endothelial bEND.3 cells. (5 experiments in duplicates). (**B**) Abundance of sCD200 in serum collected from wild-type and *Canx^−/−^* mice. (n = 3 per group). (**C**) Representative images (n = 3 views per experiment) of T-cells (green) adhered to wild-type bEND.3 monolayers cultured in the absence or presence of serum collected from wild-type and *Canx^−/−^* mice. * *p* = 0.0325, ** *p* = 0.0057, **** *p* < 0.0001. The experiment was repeated 3 times in triplicates. Blue, DAPI staining (**D**) Ratio of T-cells to wild-type bEND.3 cells in cultures incubated with serum collected from wild-type (black dots) or *Canx^−/−^* mice (red symbols). (**E**) A schematic representation of how the binding of sCD200 produced by endothelial cells to CD200R1 on T-cells antagonizes the adhesion of T-cells with endothelial cells and thereby inhibits the process of transmigration. Wild-type endothelial cells of the blood–brain barrier (left panel) produce a basal amount of CD200 and sCD200. By contrast, *Canx^−/−^* or *Fabp5^−/−^* brain endothelial cells produce elevated levels of CD200 and sCD200. The amount of sCD200 produced saturates the CD200R1 on activated T-cells, preventing them from interacting with endothelial cells, thus attenuating T-cell adhesion and transmigration across the blood–brain barrier (middle panel). The addition of excess anti-CD200 antibodies antagonizes the binding of CD200 and sCD200 produced by *Canx^−/−^* or *Fabp5^−/−^* brain endothelial cells to CD200R1 on T-cells, thus enabling them to interact with CD200 on endothelial cells and subsequently cross the blood–brain barrier (right panel).

The extracellular domain of cell surface CD200 is known to undergo proteolytic cleavage to generate a soluble form, sCD200 [25,26,27]. We therefore examined whether the abundance of sCD200 was also altered in the absence of calnexin or Fabp5. The analysis of conditioned media of *Canx*^−/−^ or *Fabp5*^−/−^ bEND.3 cells showed substantially greater concentrations of sCD200 (7.9-fold increase, *p* < 0.0001; 4.1-fold increase, *p* = 0.0003; respectively) relative to that of wild-type bEND.3 cells (Figure 4A). Consistent with this finding, a higher concentration of sCD200 (2.5-fold increase, *p* < 0.05) was found in the serum of calnexin-deficient mice compared to wild-type mice (Figure 4B). These findings illustrate that the deficiency of calnexin or Fabp5 in bEND.3 endothelial cells or the deficiency of calnexin in mice promoted an increase in the abundance of not only endothelial cell surface CD200 (Figure 1) but also sCD200 (Figure 4A,B). Importantly, the co-incubation of serum from *Canx*^−/−^ mice with wild-type bEND.3 cells drastically reduced the adhesion of activated T-cells to wild-type bEND.3 (*p* < 0.0001) (Figure 4C), consistently with the reduced number of T-cells detected in the lower chamber of transmigration assays (Figure 2). Thus, contrary to the initial expectation, the increased CD200 gene expression by bEND.3 cells upon the loss of calnexin or Fabp5 resulted in the attenuation, rather than promotion, of the adhesion of T-cells to endothelial cells (Figure 4D). Taken together, our findings demonstrate that the increased abundance of sCD200 was responsible for preventing T-cells transmigration across *Canx^−/−^* and *Fabp5^−/−^* bEND.3 cell monolayers, and that the increased production of CD200 by brain endothelial cells is the key modulator of T-cell transmigration across the blood–brain barrier in mice lacking calnexin or Fabp5 (Figure 4E).

### 2.3. CD200 in Blood Vessels in MS Brain

**Figure 5:**
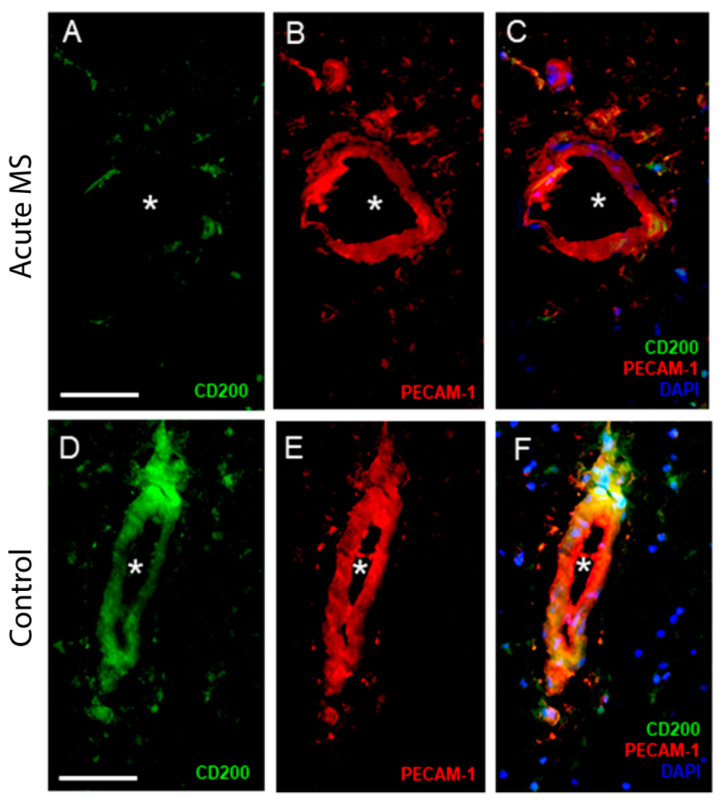
CD200 in brain endothelial cells from MS lesions. Acute MS brain lesions demonstrate low levels of CD200 (asterisk) and thus lack co-localization with PECAM-1^+^ brain endothelial cells (**A**–**C**). Unaffected brain white matter shows high CD200 abundance and co-localization with PECAM-1^+^ brain endothelial cells (**D**–**F**). Scale bar: 50 µm. Representative images of samples from 2 unaffected and 6 MS donors.

**Figure 6:**
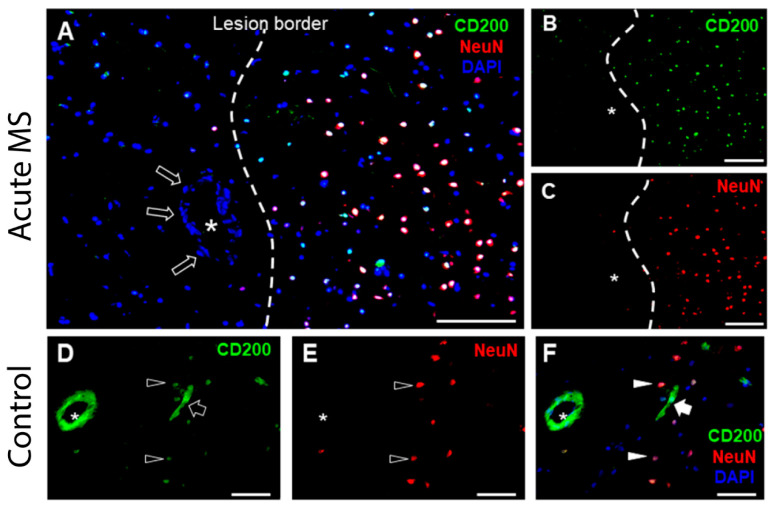
CD200 is lacking in MS lesions compared with unaffected white matter. Representative image of an MS lesion in white matter, left to the lesion border, adjacent to grey matter, right to the lesion border. (**A**–**D**) shows a juxtacortical lesion, a well-recognized type of lesion in MS. Samples were stained for CD200 (green) (**A**,**B**,**D**,**F**) and neuronal marker NeuN (red) (**C**,**E**,**F**). CD200 was not detected in the endothelial layer (open arrows in **D**,**E** and solid arrows in **F**) in the blood vessels marked with an asterisk within the lesion, while neurons showed immunopositivity for CD200 outside the lesion area (**B**). Positive immunostaining for CD200 in unaffected brain blood capillaries (open arrows) and neuronal cell bodies (open arrowheads) (**A**,**D**). Scale bar: 100 µm for **A**, 50 µm for **B**–**F**. Representative images of samples from 2 unaffected and 6 MS donors.

We then examined whether CD200 abundance was altered in brain endothelial cells from blood vessels within the brain lesions of MS patients. The histological analysis of fresh-frozen tissue sections of white matter from tissue donors without or with MS showed that CD200 abundance in PECAM-1^+^ endothelial cells was markedly reduced in samples from donors with MS (Figure 5A–C) in comparison to samples from non-affected donors (Figure 5D–F). We also analyzed white matter lesions compared to adjacent non-inflammatory white matter in tissue sections from MS donor samples. In MS white matter lesions infiltrated with macrophages, CD200 was detected in neurons (NeuN^+^ cells) (Figure 6B,C,F) and blood vessels (Figure 6E,F) from normal-appearing white matter adjacent to MS lesions. By contrast, CD200 abundance was drastically reduced in MS lesions (Figure 6A–C), which is similar to that observed after the induction of EAE in wild-type mice [24], which is consistent with the model depicted in Figure 4E.

## 3. Discussion

Calnexin is an integral ER membrane protein and molecular chaperone involved in the quality control of newly synthesized glycoproteins [14,28,29]. The whole-body inactivation of calnexin leads to myelinopathy [13] as well as resistance to EAE induction [17], an animal model of CNS inflammation [21,22]. Interestingly, we discovered that the cytoplasmic domain of calnexin interacts and forms a complex with Fabp5 [16], a member of the group of fatty acid-binding proteins [15]. Unexpectedly, Fabp5-deficient mice are also highly resistant to EAE induction [19,30], and subsequently we found that the complex formed by calnexin and Fabp5 favors T-cell transmigration across a layer of brain endothelial cells in vitro [17,20]. Thus, *Canx^−/−^* and *Fabp5^−/−^* mice are apparently protected from EAE pathology due to decreased T-cell transmigration across the blood–brain barrier [17,20].

We also found previously that the formation of the immune system in mice does not require calnexin, since the thymus and peripheral T-cell populations of calnexin-deficient mice appear normal [13]. Considering the role of calnexin as a molecular chaperone for N-linked glycoproteins [14,28,29], it was reasonable to hypothesize that the permeability of the blood–brain barrier to T-cells is attributable to an altered abundance of adhesion and/or tight junction molecules. However, we found that the abundance of proteins on the surface of *Canx*^−/−^ and *Fabp5*^−/−^ brain endothelial bEND.3 cells that are involved with leukocyte adhesion and transmigration were at similar levels to wild-type bEND.3 cells, suggesting that the basis for the resistance of calnexin-deficient and Fabp5-deficient mice to EAE induction is not associated with changes in the abundance of adhesion and/or tight junction proteins in the blood–brain barrier. Instead, the absence of calnexin or Fabp5 affects the communication between brain endothelial cells and T-cells.

The naturally occurring *Wld^S^* mouse mutant displays an attenuated course of EAE induction [23], which is coincident with observations in both *Canx*^−/−^ and *Fabp5*^−/−^ mice [17,19]. *Wld^S^* mice have been shown to overproduce CD200 [23]. CD200 is broadly expressed by cells in the CNS, including endothelial cells [6,7,8], and this led us to question whether CD200 played a role in the pathogenesis of EAE. The interaction of CD200 with the CD200R1 expressed on leukocytes and microglia triggers a regulatory signaling cascade that suppresses local inflammation and promotes a resting state in immune cells [7,9,10,31]. The brain infiltration by myeloid and lymphoid cells is highly increased in CD200-deficient mice, which points to an important role for this protein in determining blood–brain barrier permeability for leukocytes [7,12,31]. Indeed, flow cytometry analysis revealed that the loss of either calnexin or Fabp5 increased the abundance of CD200 on bEND.3 cells, coincidently with their phenotype of resistance to T-cell passage in our transmigration assay, while the preincubation of bEND.3 cells with anti-CD200 blocking antibody potently allowed T-cell passage across *Canx^−/−^ and Fabp5^−/−^* bEND.3 cells. Furthermore, the siRNA-mediated inhibition of CD200 gene expression in both *Canx^−/−^ and Fabp5^−/−^* bEND.3 cells recapitulated the loss of resistance to T-cell passage exhibited by these cells when co-incubated with the anti-CD200 blocking antibody.

In humans, CD200 is present predominantly on immune cells, but has also been shown to be prevalent on vascular endothelial cells [6,8]. During chronic inflammation, peripheral inflammatory myeloid cells (monocytes, macrophages, and dendritic cells) are present. These cells in turn can promote effector T-cell inflammatory properties such as inflammatory cytokine release and migration across the blood–brain barrier. The expression of CD200 on vascular endothelial cells may act as a checkpoint inhibitor engaging with CD200R1 on inflammatory myeloid or lymphoid cells to maintain blood–brain barrier function. Thus, the observed loss of CD200 on brain endothelial cells in MS brain lesions is expected to exacerbate the localized immune cell-mediated response. CD200 is also expressed on several cell types in CNS, including neurons, where CD200 can modulate the inflammatory properties of CD200R-expressing microglia [32]. In MS, macrophages and T-cells infiltrate lesion areas of the brain and cause damage. In this study, we found that CD200 was deficient in lesion areas but evident in non-lesion areas of MS white matter, where it can retain a protective role. Previous studies on CD200-deficient mice showed that the loss of CD200 led to an increased infiltration of T-cells and monocytes across the blood–brain barrier, assisted by increased pro-inflammatory cytokines, which adds support to the idea that CD200 gene expression by brain endothelial cells is important in acting as a gatekeeper, preventing inflammatory cells from entering CNS.

Endothelial cells with a high surface abundance of CD200 show increased binding to CD200R on T-cells in a cell adhesion assay [8,31]. Thus, the increased abundance of CD200 on *Canx^−/−^* and *Fabp5^−/−^* bEND.3 endothelial cells initially suggested that these cells would bind T-cells avidly but retain them on endothelial cell surfaces, since only few T-cells manage to migrate into the lower culture chamber in transmigration assays. Surprisingly, confocal microscopy showed substantially fewer T-cells bound to *Canx^−/−^* and *Fabp5^−/−^* bEND.3 cells despite an increased CD200 abundance on their surfaces.

It has been shown that alternate forms of CD200 exist. CD200tr is encoded by the CD200tr mRNA splice variant [33], and sCD200 is a soluble form that is produced by the proteolytic cleavage of the extracellular domain of the membrane-bound CD200 [25,26,27]. The analysis of blood samples revealed a higher concentration of sCD200 in *Canx*^−/−^ mice compared to wild-type mice. Moreover, up to 3 h co-incubation of activated T-cells with sCD200-enriched serum from *Canx*^−/−^ mice, unlike serum from wild-type mice, inhibited the adhesion of T-cells to wild-type bEND.3 cells. These findings illustrate that an increased abundance sCD200 in *Canx*^−/−^ mice contributes to antagonizing the adhesion of T-cells with endothelial cells and thereby inhibits the process of transmigration (Figure 4E).

Collectively, our data have uncovered the molecular basis for the resistance of mice deficient in either calnexin or Fabp5 to EAE induction as the antagonism of T-cell interaction with endothelial cells afforded by the increased production of sCD200. Our discovery identifies the events downstream of the Fabp5/calnexin C-tail domain complex formation at the cytosol/ER membrane interface, culminating with the increased production of sCD200, as well as CD200 itself, as novel avenues for the development of new therapeutic strategies to minimize the invasion of the CNS with potentially disease-inducing T-cells, such as in MS.

## 4. Materials and Methods

### 4.1. Mice, Human Samples, and Ethics

*Canx^−/−^* mice were generated as previously described [13]. All methods were carried out in accordance with relevant guidelines and regulations, and approved by Biosafety Officers in the Health, Safety, and Environment Office at the University of Alberta. All animal experiments were carried out according to the University of Alberta Animal Policy and Welfare Committee and the Canadian Council on Animal Care Guidelines. The approval for use of mice in research was granted by the Animal Care and Use Committee for Health Sciences, University of Alberta ethics review committee (Permit AUP297).

Post-mortem human brain tissue from both MS (n = 6) and normal control cases without CNS disease (n = 2), and the associated clinical and neuropathological data, were obtained with ethical approvals from tissue banks. Samples were supplied by the MS Society Tissue Bank, multicenter ethics number 08/MRE 09/31; and from the NeuroResource tissue bank and Queen Square Brain Bank for Neurological Disorders, University College London (UCL) Queen Square Institute of Neurology, both with Tissue Bank National Health Services (NHS) Research Ethics Committee approval 18/LO/0721 from the London—Central Research Ethics Committee. Supplemental Table S1 shows the characteristics of the study population.

### 4.2. Mouse Brain Endothelial (bEND.3) Cells

The bEND.3 cell line (CRL-2299) was purchased from ATCC (Manassas, VA, USA) and cultured under the recommended conditions [34]. Molecular genotyping revealed the sex of these cells as male [17]. The CRISPR/Cas9 gene editing technique was used for silencing of the calnexin and Fabp5 genes [17,18,19,20]. Individual clones were isolated from the cell population transfected with single guide RNAs and identified by PCR and immunoblot analyses, to confirm the successful inactivation of the calnexin or Fabp5 genes [20]. For PCR analysis, genomic DNA from the modified region was amplified using specific DNA primers and sequenced to confirm gene editing [17]. Wild-type, *Canx^−/−^,* and *Fabp5^−/−^* bEND.3 cells were cultured in DMEM media (Thermo Scientific, Waltham, MA, USA) supplemented with 10% fetal bovine serum (FBS) at 37 °C and 5% CO_2_ until reaching 90% confluency. Cells were treated overnight with a combination of 60 ng/mL IFN-γ (eBioscience, San Diego, CA, USA) and 3 ng/mL TNF-α ((Invitrogen, Waltham, MA, USA) and recovered with TrypLE Express™ (Thermo Scientific, Waltham, MA, USA) for further analysis.

### 4.3. Flow Cytometry

A combination of anti-mouse monoclonal antibodies for extracellular markers was used (Supplementary Table S1). Cells were first treated with Live/Dead aqua™ viability dye (0.5 μL/10^6^ cells) (Life Technologies, Waltham, MA, USA) in PBS, washed with PBS followed by a blocking buffer (PBS, 1% BSA and 0.5 mM EDTA), and stained with the appropriate fluorochrome-conjugated antibodies. All monoclonal antibodies were titrated to determine the optimal usage concentration. After a 15 min incubation at room temperature in the dark, cells were washed with a buffered solution (PBS, 1% BSA, and 0.5 mM EDTA) and analyzed. For each experiment, a minimum of 10,000 live/single events were recorded using the LSRFortessa™ X-20 Flow cytometer (BD Biosciences Pharmingen, Franklin Lakes, NJ, USA). Data analysis was performed using FlowJo™ vX for PC BD Biosciences Pharmingen, Franklin Lakes, NJ, USA).

### 4.4. Quantitative RT-PCR

Total RNA was extracted from cells using the RNeasy Plus™ Kit (Qiagen, Hilden, Germany) and stored at −80 °C until used. The concentration of RNA was measured using a NanoDrop™ (Thermo Scientific) followed by reverse transcriptase reaction using the iScript reverse transcription kit™ (Bio-Rad, Hercules, CA, USA) and gene amplification with the PerfeCTa SYBR Green Supermix™ RT-PCR kit (Quantabio Beverly, MA, USA). DNA primers were designed using the NCBI primer design online tool (https://www.ncbi.nlm.nih.gov/tools/primer-blast/, accessed on 27 June 2024). Quantitative RT-PCR analysis was performed using a Rotor-Gene™ Q (Qiagen). The relative quantification was obtained by normalizing each target gene expression to the 18S rRNA endogenous control expression using the 2^−ΔΔCt^ method. The following primers were used for qPCR: for 18S rRNA, 5′-GCC GCT AGA GGT GAA ATT CT-3′ and 5′-TCG GAA CTA CGA CGG TAT CT-3′, and for CD200, 5′-TAA TCC AGC CTG CCT ACA AAG-3′ and 5′-CAG CCC TCA TCT TCC AAT GT-3′.

### 4.5. Soluble CD200 (sCD200) ELISA

Wild-type, *Canx^−/−^*, and *Fabp5^−/−^* bEND.3 cells were seeded into 6-well plates at 80% confluency in 3 mL of DMEM medium. After 48 h of incubation, the cell supernatant from the confluent plates was collected and then centrifuged to remove remaining cells. The supernatants were transferred to new tubes and the samples were frozen at −80 °C until analysis. On the day of analysis, the samples were thawed at room temperature and mixed by vortexing, and 100 µL of each sample was used for the quantification of sCD200, using the mouse CD200 ELISA Kit Picokine™ (Boster Bio Cat#EK1185, Pleasanton, CA, USA) as per the manufacturer-recommended protocol.

### 4.6. Adhesion Assay

On Day 0, wild-type, *Canx^−/−^*, and *Fabp5^−/−^* bEND.3 cells were seeded in duplicates into 12-well removable chambers (Ibidi Cat. #81201). On the same day, freshly isolated splenocytes were seeded in 6-well plates coated with 2 µg/mL of anti-CD3 (clone: OKT3, BioLegend, San Diego, CA, USA) and 2 µg/mL of soluble anti-CD28 (clone: 37.51, BioLegend, San Diego, CA, USA) antibodies in 3 mL of DMEM 10% FBS, 1% penicillin/streptomycin. On Day 2, the bEND.3 cells were treated with 60 ng/mL IFN-γ (eBioscience, San Diego, CA, USA) and 3 ng/mL TNF-α (Invitrogen™) inflammatory cytokines for 18 h. On Day 3, the bEND.3 cells were treated with 20 µg/mL of anti-CD200 blocking antibody (clone: OX90, BioLegend) or Ultra-LEAF™ isotype control antibody (BioLegend) and incubated for 3 h. The activated splenocytes were labelled with the CellTrace™ CFSE Cell Proliferation Kit for flow cytometry (Molecular Probes, Eugene, OR, USA), and 10^4^ labelled cells were added to each well containing the bEND.3 cell monolayers. After 3 h of incubation, the chambers were removed from the glass slides and the samples were processed for microscopy. Slides were fixed with 4% paraformaldehyde in PBS for 10 min at room temperature. After washing with PBS, a block/permeabilization buffer (PBS 0.3% TWEEN 20, 5% BSA) was added for 10 min incubation at room temperature. After washing 3 times with PBS, cells were stained with 0.4 U/mL of Phalloidin CF633™ (Biotium, Fremont, CA, USA) diluted in PBS for 40 min at room temperature, followed by incubation with 4′,6-diamidino-2-phenylindole (DAPI) at 1 µL/mL in PBS for 5 min at room temperature. Slides were washed 3 times with PBS followed by the addition of ProLong™ Diamond Antifade Mountant (Thermo Scientific).

For the treatment of wild-type bEND.3 with mouse serum, cells were seeded in 12-well removable chambers. On Day 2, the cells were treated with 60 ng/mL IFN-γ and 3 ng/mL TNF-α inflammatory cytokines for 18 h. On Day 3, the culture medium was replaced with 150 µL of RPMI containing 50 µL of mouse serum collected from either wild-type or *Canx*^−/−^ male mice (n = 3 for each genotype). After 3 h, 10^5^ leukocytes (isolated from a total of 3 male wild-type C57BL/6J mice and labelled with CFSE as described previously [17,20]) were added into each well in a total volume of 20 µL followed by confocal microscopy. Images were captured with a Laser Scanning Confocal Microscope Leica TCS SP5 (Leica Microsystems, Wetzlar, Germany) and analyzed with Image J software v1.54f (NIH) for the ratio of CFSE-labelled lymphocytes to the bEND.3 nuclei count.

### 4.7. siRNA-Mediated Gene Silencing Experiments and Transmigration Assays

On Day 0, wild-type, *Canx^−/−^,* and *Fabp5^−/−^* bEND.3 cells were seeded on the membrane of Corning HTS Transwell™ 96-well permeable supports with 5.0 μm pore size (MilliporeSigma, Burlington, MA, USA) [17,20]. On Day 1, the bEND.3 cells were incubated with 1 µM of Accell SMARTPool siRNA™ targeting CD200 (Dharmacon^TM^ Cat #058472-00-0050, Lafayette, CO, USA) or non-targeting Accell siRNA™ (Dharmacon^TM^ Cat. #D-001910-0X) mixed with 2.2 µg/mL polyethyleneimine in reduced serum medium (OPTI-MEM™, Thermo Scientific, Waltham, MA, USA) for 72 h. Cells that were not treated with siRNAs were also maintained in reduced serum medium with polyethyleneimine. On Day 2, the cells were treated with 60 ng/mL IFN-γ (eBioscience) and 3 ng/mL TNF-α (Invitrogen™) inflammatory cytokines for 18 h. For antibody-mediated CD200 blocking, bEND.3 cells were incubated with 20 µg/mL of anti-CD200 blocking antibodies (clone: OX90, BioLegend) or Ultra-LEAF™ isotype control antibodies (BioLegend) for 18 h.

For the transmigration assay, on Day 0, splenocytes were isolated from male wild-type C57BL/6J mice (total of n = 8) and seeded in 6-well plates coated with 2 µg/mL of anti-CD3 (clone: OKT3, BioLegend), in DMEM 10% FBS and 1% Pen/Strep with 2 µg/mL of soluble anti-CD28 (clone: 37.51, BioLegend) antibodies to activate and proliferate T-cells. On Day 3, cells were labelled with the CellTrace™ CFSE dye using CellTrace™ CFSE Cell Proliferation Kit for flow cytometry (Molecular Probes, Eugene, OR, USA), seeded in the upper chamber (10^4^ cells per well), over the bEND.3 cell monolayers and incubated for 4 h. The labelled cells that transmigrated into the lower chamber were then counted using a BD Accuri C6™ Flow cytometer (BD Biosciences Pharmingen, Franklin Lakes, NJ, USA). Migrated cells were gated as lymphocytes by SSC and FSC characteristics (Supplementary Figure S2A). The percentage of T-cells (CD3^+^ cells) found in the SSC/FSC lymphocyte gate was enriched from ≈26% (before anti-CD3/CD28 treatment) to ≈97% (after 3 days of antibody treatment) (Supplementary Figure S2B,C), hereafter referred to as activated T-cells. CD200R1 was confirmed in the surface of activated cells (Supplementary Figure S2D).

### 4.8. Enzymatic Digestion of Brain Tissue

Brains were isolated from male wild-type (n = 7) and male *Canx*^−/−^ mice (n = 7) and then perfused with PBS as described previously [17]. The tissue was ground using a 100 µm nylon strainer with a syringe plunger and recovered into 4 mL of 1 mg/mL papain (Worthington Biochemical, Lakewood, NJ, USA) in PBS and 25 μL of DNAse I (Thermo Scientific) at 5 mg/mL were added into each tube. The samples were incubated at 37 °C for 15 min with repeated agitation and then washed with complete DMEM medium. After treatment with a red blood cell buffer solution (150 mM NH_4_Cl, 10 mM KHCO_3_, 0.1 mM EDTA, pH 7.3), the cells were washed with PBS, followed by enrichment on a 30% Percoll (MilliporeSigma, Burlington, MA, USA) gradient to remove myelin [35]. Cells were washed with PBS and labelled for flow cytometry analysis. Endothelial cells were identified as CD11b^−^PECAM-1^+^ (Supplementary Figure S3).

### 4.9. Histology and Immunohistochemistry of Human Brain Tissues

For immunofluorescence staining, post-mortem snap-frozen brain tissue samples were stained with specific antibodies as indicated in Supplemental Table S2. Glass-mounted tissue sections (10 µm thick) were fixed in 4% paraformaldehyde and incubated sequentially with primary and secondary antibodies in PBS at 1:100 concentration. Autofluorescence Eliminator Reagent (MilliporeSigma, Cat. #2160, Burlington, MA, USA, Burlington, MA, USA) was added to the tissue for 5 min and washed off with 70% ethanol. Tissue sections were washed, rehydrated, and mounted in anti-quenching fluorescent mounting medium with DAPI-ProLong™ Gold anti-fade mountant (Thermo Scientific), and stored at 4 °C in the dark for subsequent microscopic examination. Immunofluorescence detection was observed using air and oil immersion objectives, and high-resolution images were captured using a Leica DM4000 B LED fluorescence microscope (Leica Microsystems, Wetzlar, Germany) equipped with a black and white digital camera.

### 4.10. Statistics

The data were analyzed using GraphPad Prism™ v7 (GraphPad Software Inc., La Jolla, CA, USA). The normality of data distribution was determined using the Kolmogorov–Smirnov test. The differences between group means were determined by one-way analysis of variance (ANOVA for multiple groups or Student’s *t*-test for two groups), with the Mann–Whitney, Dunn’s or Tukey post-hoc tests where appropriate. Statistical significance was assumed when *p* < 0.05.

## Data Availability

Data will be made available upon reasonable request.

## Supplementary Materials

The following supporting information can be downloaded at: https://www.mdpi.com/article/10.3390/ijms25179262/s1.

## Abbreviations

Canx	Calnexin
CD200R1	CD200 receptor 1
CNS	Central Nervous System
EAE	Experimental Autoimmune Encephalomyelitis
ER	Endoplasmic Reticulum
Fabp5	Fatty acid binding protein 5
ICAM-1	Intercellular Adhesion Molecule 1
JAM-A	Junctional Adhesion Molecule-A
MS	Multiple Sclerosis
PECAM-1	Platelet Endothelial Cell Adhesion Molecule-1
sCD200	soluble CD200
Wld	Wallerian degeneration
VCAM-1	Vascular Adhesion Molecule-1
ZO-1	Zonula Occludens-1
