# Supplementary material for: Endothelial Cell-Derived Soluble CD200 Determines the Ability of Immune Cells to Cross the Blood–Brain Barrier
Source: Int J Mol Sci. 2024 Aug 27;25(17):9262. doi: 10.3390/ijms25179262 (PMC11395061; doi:10.3390/ijms25179262)
Supplement: Supplementary file 1 [file ijms-25-09262-s001.zip › ijms-3105837-supplementary.pdf]

Supplemental Table S1

Antibodies used for flow cytometry analyses.

Target	Clone	Fluorochrome	Source
CD200	OX-90	APC	BioLegend
ICAM-I	EBioKAT-1	PE	eBioscience
VCAM-I	429	FITC	eBioscience
P-SELECTIN	Psel.KO2.12	FITC	eBioscience
ZO-1	POLYCLONAL	FITC	Bioss
JAM-A	H202-106	DL755	Novusbio
PECAM-1	390	FITC	BioLegend
CD11b	M1/70	SB-645	Invitrogen

Supplemental Table S2

Antibodies used for immunofluorescence staining of CNS tissue.

**Target**	**Clone**	**Host Species**	**Dilution used**	**Source**	**Product number**
CD200	OX-2	Rabbit	1:100	Abcam	Ab203887
PECAM-1	P2B1	Mouse	1:100	Abcam	Ab24590
NeuN	1B7	Mouse	1:100	Abcam	Ab104224

**SECONDARY ANTIBODIES**	**Clone**	**Fluorochrome**	**Dilution used**	**Source**	**Product number**
Goat anti-rabbit	IgG (H+L) Highly Cross-Adsorbed	Alexa Fluor 488	1:100	ThermoFisher	A11034
Goat anti-rabbit	IgG (H+L) Cross-Adsorbed	Alexa Fluor 647	1:100	ThermoFisher	A21244
Goat anti-mouse	IgG (H+L) Cross-Adsorbed	Alexa Fluor 647	1:100	ThermoFisher	A21235

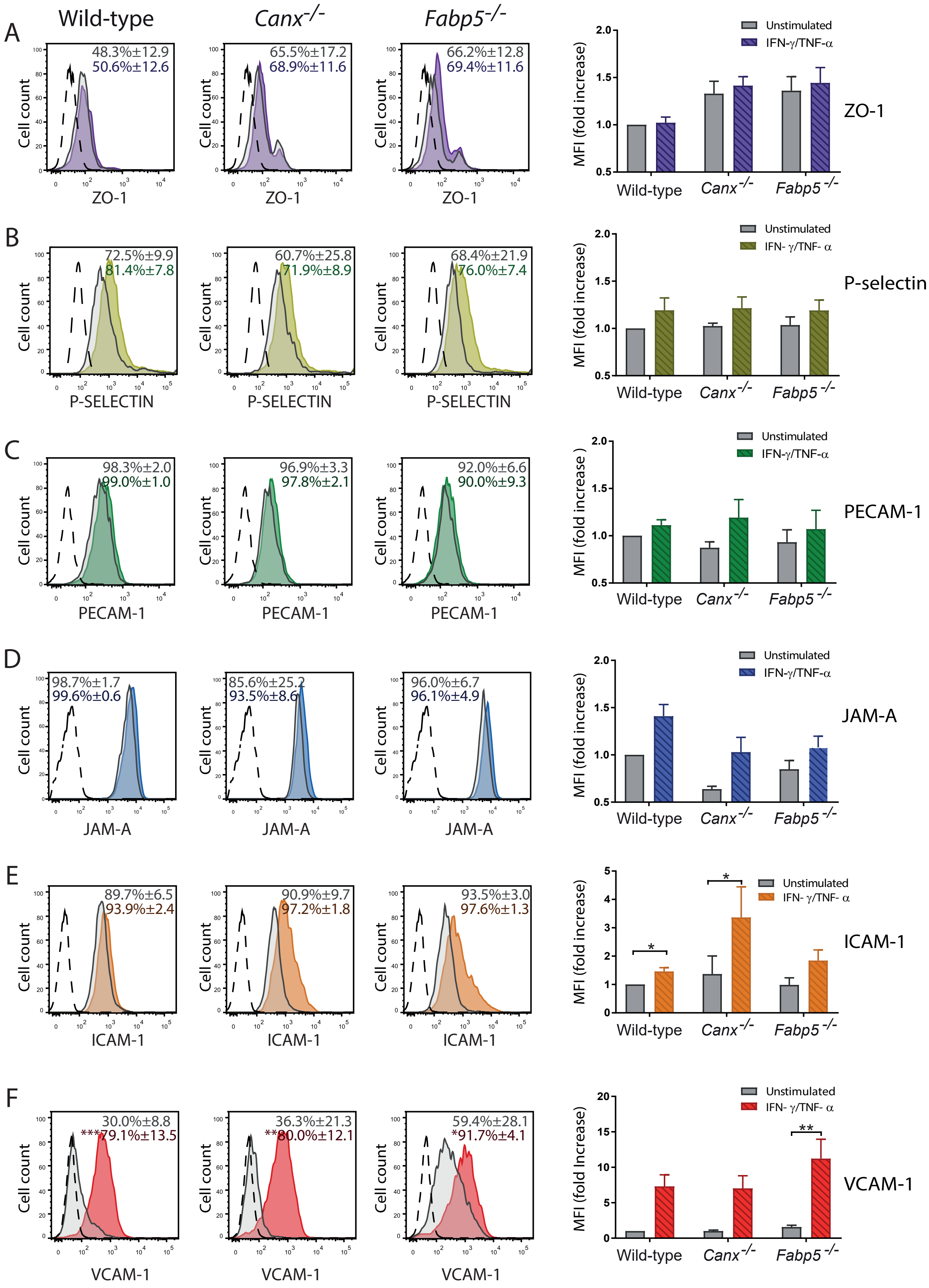

Supplemental Figure S1

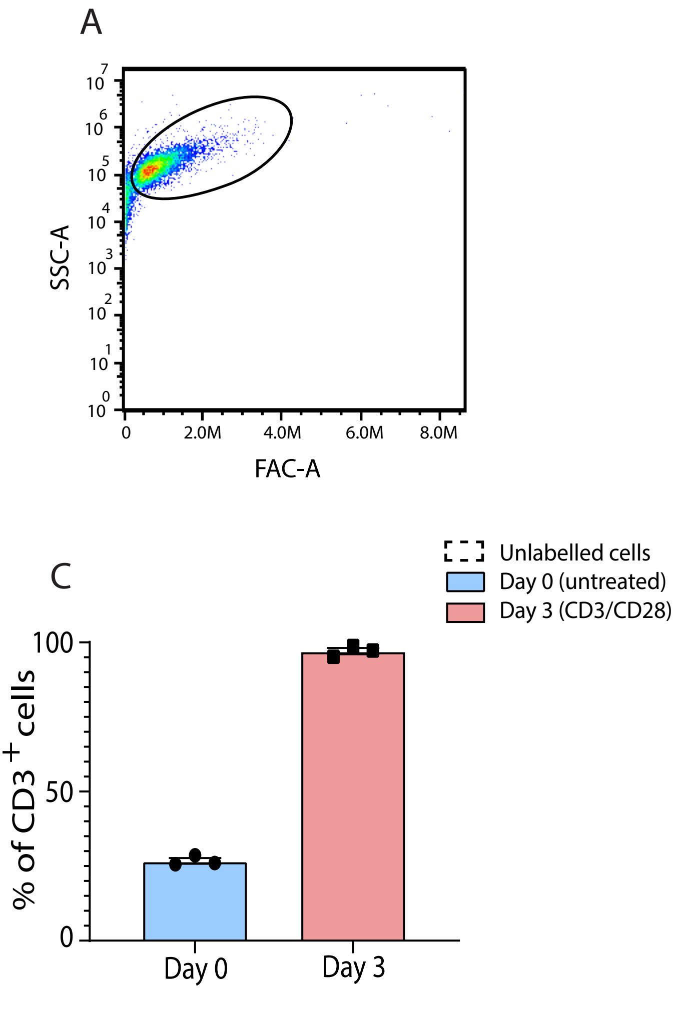

Supplemental Figure S2

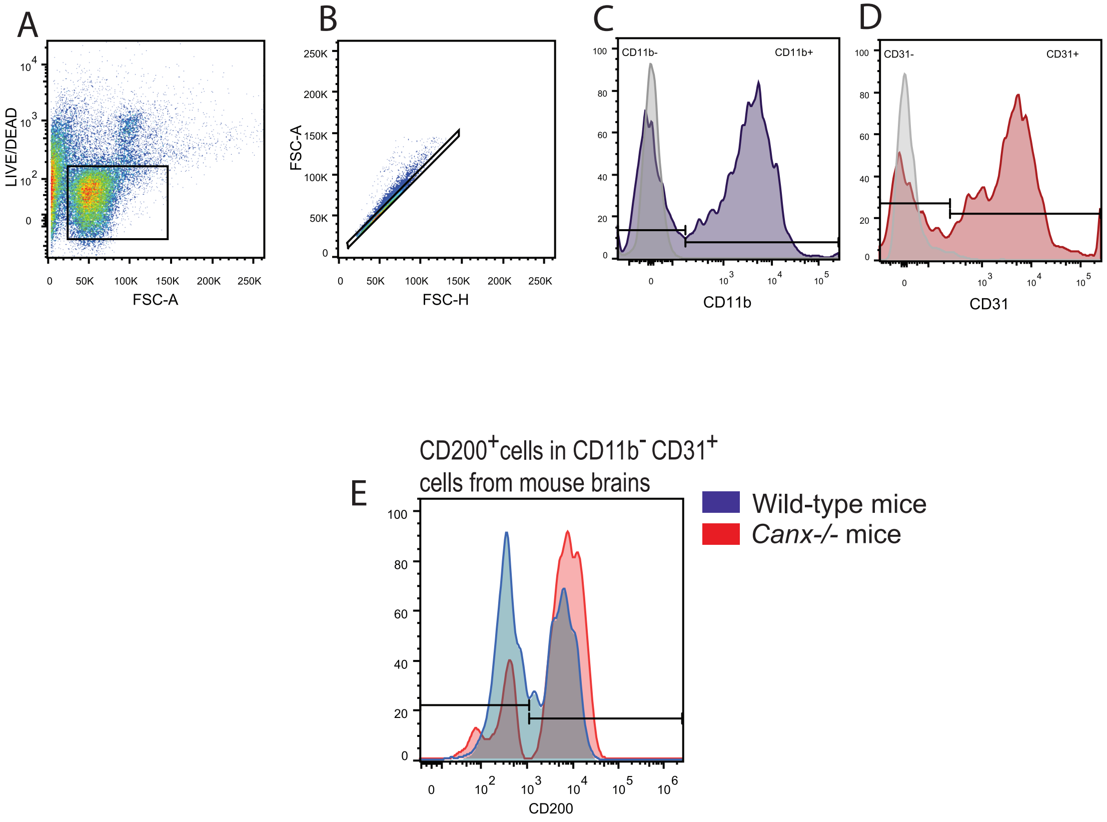

Supplemental Figure S3
